# Mesenchymal Stem Cells: Rising Concerns over Their Application in Treatment of Type One Diabetes Mellitus

**DOI:** 10.1155/2015/675103

**Published:** 2015-10-20

**Authors:** Seyed Jafar Hashemian, Marjan Kouhnavard, Ensieh Nasli-Esfahani

**Affiliations:** ^1^Diabetes Research Center, Endocrinology and Metabolism Clinical Sciences Institute, Tehran University of Medical Sciences, Tehran, Iran; ^2^Endocrinology and Metabolism Research Center, Endocrinology and Metabolism Research Institute, Tehran University of Medical Sciences, Tehran, Iran

## Abstract

Type 1 diabetes mellitus (T1DM) is an autoimmune disorder that leads to beta cell destruction and lowered insulin production. In recent years, stem cell therapies have opened up new horizons to treatment of diabetes mellitus. Among all kinds of stem cells, mesenchymal stem cells (MSCs) have been shown to be an interesting therapeutic option based on their immunomodulatory properties and differentiation potentials confirmed in various experimental and clinical trial studies. In this review, we discuss MSCs differential potentials in differentiation into insulin-producing cells (IPCs) from various sources and also have an overview on currently understood mechanisms through which MSCs exhibit their immunomodulatory effects. Other important issues that are provided in this review, due to their importance in the field of cell therapy, are genetic manipulations (as a new biotechnological method), routes of transplantation, combination of MSCs with other cell types, frequency of transplantation, and special considerations regarding diabetic patients' autologous MSCs transplantation. At the end, utilization of biomaterials either as encapsulation tools or as scaffolds to prevent immune rejection, preparation of tridimensional vascularized microenvironment, and completed or ongoing clinical trials using MSCs are discussed. Despite all unresolved concerns about clinical applications of MSCs, this group of stem cells still remains a promising therapeutic modality for treatment of diabetes.

## 1. Introduction

Type 1 diabetes mellitus (T1DM) is an autoimmune disease leading to beta cell destruction and lowered insulin production [1]. Insulin administration, as the standard treatment strategy for type 1 diabetes, cannot exactly mimic the physiologic secretion of insulin in the body [2].

To date, pancreatic and islet transplantation have been shown to be relatively effective therapeutic options [3, 4]. However, complications associated with the transplantation procedure, the need for life-long immunosuppressant therapy, with its adverse side effects, and the difficulty of obtaining transplant material and organ donations have restricted these treatment modalities [5].

Therefore, looking for other therapeutic options which can resemble islet cell function with limited complications seems crucial. Among all kinds of stem cells, mesenchymal stem cells (MSCs) have been shown to be an interesting therapeutic option due to their immunomodulatory properties and their potential for in vitro differentiation into insulin-secreting cells.

This review summarizes the main features of mesenchymal stem cells as well as their use in the treatment of diabetes mellitus.

## 2. History and Sources

Fibroblast-like cell colonies from bone marrow were first isolated by Friedenstein and his colleagues in 1976 [6]. Later on, Caplan called these cells “mesenchymal stem cells” (MSCs) based on their features [7]. Bone marrow-derived MSCs (BMMSCs) are multipotent, nonhematopoietic stromal cells capable of adhering to cell culture surface as well as having long-term self-renewal and multilineage differentiation capacities [8–10]. However, the term “multipotent mesenchymal stromal cells” is currently being used for this population of cells [11].

MSCs can also be isolated from various tissues and organs such as placenta, cord blood, umbilical cord Wharton's jelly, pancreas, and adipose tissue [12–22].

## 3. Differentiation Capacities

A large number of studies have demonstrated that bone-marrow-derived MSCs have the potential to differentiate into mesodermal, ectodermal, and endodermal tissues including bone [23, 24], muscle [25, 26], neurons [27], hepatocytes [28] as well as skin [29–34], cardiomyocytes [35–38], and other tissues [9, 39–42]. In addition to angiogenesis promotion, several experimental studies have revealed that MSCs are able to differentiate into insulin-producing cells (IPCs) as well [43–48].

## 4. Markers

To date, there is no specific marker or group of markers to identify MSCs. As a result, this group of cells has been identified according to the combination of their surface markers and functional characteristics. Generally, MSCs express Stro-1 [49–51], CD105 (SH2) [52], CD73 (SH3/4) [53], CD90, CD146, and CD200 [54], in addition to some cell adhesion molecules including integrins (*α* and *β*) [55], intercellular adhesion molecule-1 and intercellular adhesion molecule-2 (ICAM-1 and ICAM-2), vascular cell adhesion molecule-1 (VCAM-1), lymphocyte function-associated antigen-3 (LFA-3), CD72, and activated leucocyte-cell adhesion molecule (ALCAM) [8, 56–61].

The Mesenchymal and Tissue Stem Cell Committee of the International Society for Cellular Therapy (ISCT) introduces minimal criteria to standardize human MSCs definition. First, MSCs must be adherent to the surface of standard plastic culture vessels. Second, MSCs must express CD105, CD73, and CD90, but they do not express CD45, CD34, CD14 or CD11b, CD79a or CD19, and HLA-DR surface molecules [8, 62, 63]. Third, MSCs must be able to differentiate into osteoblasts, adipocytes, and chondroblasts in vitro [64].

In other species, MSCs have different patterns of surface antigens expression. However human MSCs from different sources do not present the same surface antigens expression patterns, which makes it difficult to compare the studies in this regard, but minimal criteria of ISCT are common in all of them.

## 5. Secretive Properties

### 5.1. Cytokines and Growth Factors

It has been shown in different studies [65–70] that MSCs naturally synthesize various cytokines and growth factors which are mainly influenced by local microenvironments around them [71, 72] including M-CSF, IL-6, IL-11, IL-15, stem cell factor (SCF) [73], insulin-like growth factor-1 (IGF-1), vascular endothelial growth factor (VEGF), transforming growth factor-*β* (TGF-*β*), and hepatocyte growth factor (HGF) [74]. These trophic mediators not only promote the survival of surrounding cells [74] but also play important roles in MSCs regenerative/regulatory properties both in vitro and in vivo [67, 70, 75–77].

In Berman et al.'s study, they cotransplanted allogeneic MSCs intraportally with islets in cynomolgus diabetic monkeys. The results showed that MSCs significantly enhanced islet function and engraftment in one month after transplantation. They concluded that MSCs can provide immunomodulatory, revascularization, and regenerative cytokines [78].

In another study, coculture of MSCs with human islets from cadaveric donor improved islet's insulin secretory function in vitro. The authors believed that increased amount of trophic cytokines secreted by MSCs resulted in this improvement [79, 80].

With respect to these properties, further studies must be done to understand the underlying mechanisms and clarify the role of various growth factors in beta cell neogenesis and regeneration.

### 5.2. MSCs-Derived Microvesicles

Microvesicles (MVs) are microparticles for cell to cell communication released from different cell types, as well as from MSCs [81–83]. MVs are released to the extracellular space throughout budding and blebbing of plasma membrane [81, 84–87]; therefore they have surface characteristics of the MSCs such as CD29, CD44, and CD73 [88]. They also contain biologically active molecules such as proteins, lipids, mRNA, and microRNA [84, 89]. When MVs are transferred to recipient cells via membrane fusion or internalization, the cargo can alter the transcription, proliferation, and immunoregulation of target cells, resulting in functional and phenotypic changes [87, 88, 90]. MVs functions are specific regarding cellular source [87]. Recent findings indicated that MSCs-derived MVs (MSCs-MVs) have regenerative effects in several disease models [88, 91, 92]. In Favaro et al.'s study, they cocultured MSCs-MVs with type 1 diabetic patient's peripheral blood mononuclear cells (PBMCs). They showed that IFN*γ* level decreased in stimulated PBMCs and TGF-*β*, IL-10, IL-6, and prostaglandin E2 (PGE2) levels increased. Furthermore, MVs increased FoxP3+ regulatory T cells (Tregs) population in stimulated PBMCs [91]. Another study also demonstrated that MSCs-MVs can induce tolerogenic phenotype through induction of programmed death-ligand 1 (PD-L1), TGF-*β*, IL-10, and Tregs [93]. Although MSCs-MVs could represent a new therapeutic strategy, in clinical application of MVs, some issues such as persistency of the biologic effects, disease specificity, homing, and biodistribution must be clarified.

## 6. MSCs Application in Experimental Studies and Differentiation to IPCs

### 6.1. Bone Marrow

Bone marrow is an important source of easily accessible adult stem cells, and bone marrow transplantation is considered to be effective for the treatment of autoimmune type 1 diabetes. However, there is a great debate on the fate of transplanted bone marrow stem cells (BMCs) [94].

In 2003, Ianus et al. demonstrated that grafted mouse BMMSCs could differentiate into endocrine pancreatic cells in vivo spontaneously [95]. In contrast, additional studies failed to support these findings [96–98].

In other words, there was no evidence of bone marrow cells becoming IPCs in the pancreas of the recipient mice transplanted with GFP (green fluorescent protein) expressing bone marrow. However, it was difficult to rule out the possibility that the GFP gene was inactivated or that GFP-labeled cells were destructed as they engrafted into islets [99]. Moreover, this discrepancy might be due to diabetes etiology and/or stage as well as differences in mouse strains, mouse models, and experimental designs among research groups [100].

Other studies showed that both mouse [101] and human [99] BMMSCs ameliorated hyperglycemia in streptozotocin (STZ)-treated mice by inducing regeneration of endogenous pancreatic islets.

However, the exact mechanisms underlying the therapeutic effects of these cells have not been clearly defined. Some studies suggest that transdifferentiation of MSCs into IPCs might partly play a role in relieving hyperglycemia [95, 102–104].

On the other hand, this idea has been contradicted in another study [96] or attributed to cell fusion [105]. Xu et al. also hypothesized that MSCs transplantation into diabetic animals may prevent apoptosis of injured pancreatic beta cells and enhance regeneration of endogenous progenitor cells through paracrine actions such as angiogenic, cytoprotective, anti-inflammatory, mitogenic, and antiapoptotic effects [65].

It has been also reported that manipulation of culture conditions could induce low levels of insulin in rodent BMMSCs [102, 106–108]. However, the stability of this phenotype was unclear.

In 2009, Chang et al. transplanted male porcine BMMSCs to diabetic female pigs by direct injections into pancreas. As a result, insulin levels increased from day 14. The histological analysis revealed that newly formed islets were smaller than normal islets. In the same study, it was shown that MSCs adopt beta cell fate in diabetic pancreatic microenvironment without obvious immune rejections [109].

Gabr and colleagues differentiated human BMMSCs (hBMMSCs) into IPCs from healthy and diabetic donors. Differentiation was carried out in a 3-stage procedure with glucose-rich medium containing several activation and growth factors. Results did not show apparent differences in the rate of growth or differentiation between cells from the same donor or between cells from different donors with or without diabetes. IPCs expressed transcription factors and genes of pancreatic hormones similar to pancreatic islets. Their in vivo study in nude diabetic mice resulted in the control of their diabetic status for 3 months [110].

In a recent study, Ouyang et al. induced rat BMMSCs to differentiate into IPCs with various growth factors and small molecules through 3-step protocols. They concluded that, in their comparative study, aminopyrrole derivative compound XW4.4 revealed the best activity among them and differentiated cells exhibited morphological and functional characteristics of IPCs and expression of pancreatic endocrine cell marker genes [111].

### 6.2. Adipose Tissue

Adipose tissues isolated from human lipoaspirates are also called adipose-derived stromal cells (ADSCs) with several priorities over other sources in the way that they can be accessed easily in large amounts and possess differentiation capacities similar to BMMSCs [112].

Moreover, several experimental studies have revealed that ADSCs even have greater proliferation, differentiation, and immunomodulatory potencies compared with BMMSCs [113–117].

In the studies by Timper et al. [118] and Eberhardt et al. [119] human adipose tissue-derived MSCs (AD-MSCs) from four donors were expanded in a fibroblast growth factor-containing medium. These cells expressed stem cell markers including Isl1 mRNA, which is essential for the generation of pancreatic islet cells. Moreover, they observed upregulation of transcription factors Ipf1, Isl1, and Ngn3 and islet gene insulin, glucagon, and somatostatin, in addition to the expression of C-peptide in differentiated cells.

Chandra et al. showed that AD-MSCs from murine epididymis could differentiate into insulin-producing aggregates under a ten-day inductive protocol. Differentiated cells expressed PDX1, Ngn3, NeuroD, Pax4, Glut2, and secreted insulin and C-peptide in accordance with glucose levels. Secretory granules in the cells cytoplasm were confirmed by electron microscopy. Normoglycemic state was restored 2 weeks after intraperitoneal transplantation into diabetic mice [120].

In a recent study by Karaoz et al., it was demonstrated that AD-MSCs differentiate into IPCs after 38-day coculture with islet cells. Insulin and C-peptide production were confirmed by ELISA and immunostaining. After cotransplantation of IPCs and islet cells under kidney capsule, hyperglycemic state was recovered in diabetic rats. Moreover, it was revealed that combination of differentiated AD-MSCs and islet cells resulted in better recovery from diabetes compared to islets transplants alone or cotransplantation of islets and differentiated BMMSCs [121].

Taken together with the advantages that harvesting and producing AD-MSCs appear to be more practical and less invasive in humans compared with BMMSCs, this population of MSCs can be considered as an alternative source of IPCs.

### 6.3. Human Placenta-Derived MSCs

In 2010, for the first time, Kadam et al. [45] introduced Human Placenta-Derived MSCs (hPDMSCs) as another alternative source for cell replacement therapy in diabetes.

They showed that hPDMSCs isolated from human chorionic villi of full-term placenta can form islet-like cell clusters (ILCs) on stepwise exposure to serum-free defined media containing specific growth factors and differentiating agents.

Differentiated ILCs were found to express human insulin, glucagon, and somatostatin by immunocytochemistry. Transplantation of hPDMSCs or ILCs derived from hPDMSCs in STZ-induced diabetic mice led to restoration of normoglycemia. Their results demonstrated reversal of hyperglycemia by undifferentiated hPDMSCs and ILCs derived from hPDMSCs.

However, to evaluate hPDMSCs characteristics and their differentiation potentials, more studies should be carried out before their application in clinical trials.

### 6.4. Human Cord Blood

Human umbilical cord blood (UCB) as another source of MSCs has the potential to develop into IPCs. This issue has been indicated in several in vivo studies [122, 123].

Human UCB mononuclear-cell injections into the orbital plexus of obese B6.Y Lep(ob) mice, with spontaneous development of type 2 diabetes, improved blood glucose levels and survival rates and led to normalization of glomerular hypertrophy and tubular dilatation [122]. Furthermore, improved glycaemic profiles associated with histological improvement of insulitis were obtained after intravenous (IV) administration of hUCB-MSCs to 25 NOD type 1 diabetic mice with insulitis [123]. Later on, Yoshida et al. [124] investigated the in vivo capacities of hUCB-derived cells to generate IPCs. Thus, they transplanted hUCB cells into NOD/SCID-*β*_2_m^null^ mice and as a result they found IPCs of human origin in recipient pancreatic islets. However, the number of hUCB cells that transdifferentiated into IPCs and the rate of such an event need to be more investigated. Comparing the results of two studies by Bieback et al. [125] and Koblas et al. [126] the proportion of IPCs derived from hUCB per total number of islet cells was less than that of BM-derived IPCs. However, under diabetic conditions, the demand for the neogenesis of IPCs might increase and the rate of hUCB cells differentiation could become higher in order to compensate for the regeneration of beta cell mass. Moreover, the stem cell type in hUCB responsible for generation of IPCs remains unclear. Since MSCs isolated from cord blood [125] and hUCB-derived USSCs (unrestricted somatic stem cells) have rather the same cell markers and properties as MAPCs (multipotent adult progenitor cells) [126], it should be considered that MSCs might account for the differentiation of hUCB cells towards a beta cell phenotype.

The presence of mesenchymal stem or progenitor cells in hUCB was also indicated by other studies. They revealed that mononuclear adherent cells, isolated from UCB, display a BMMSCs-like immunophenotype and differentiation capacities [19, 115, 127]. Pessina et al. demonstrated that UCB cells exhibit genes essential for differentiation into pancreatic endocrine tissue (Isl1, PDX1, Pax4, and Ngn3), after culture in a medium supplemented with no specific cytokines or growth factors except fetal calf serum [127].

Prabakar et al. differentiated UCB-MSCs towards glucose-responsive IPCs by a stepwise culture protocol. They reported that differentiated cells express pancreas-specific transcription factors such as PDX1, Nkx6.1 and Isl1, Ngn3, and NeuroD1. UCB-MSCs-derived IPCs released insulin and C-peptide in response to a glucose challenge both in vitro and in vivo [128].

Since UCB-MSCs are available rather in large amounts, possess low risk for immune rejection [129], and have increased capacities for expansion and differentiation into IPCs, this source of MSCs can be considered as an alternative option for treatment of diabetes [130].

### 6.5. Wharton's Jelly MSCs

Not only do MSCs in hUCB have the potential to differentiate into IPCs, but also MSCs residing in umbilical cord Wharton's jelly (WJ) share the same capability.

In 2008, Chao et al. [131] could successfully differentiate WJ-MSCs into IPCs through a four-step protocol. They also transplanted the IPCs into the liver of the diabetic mice. The authors demonstrated the expression of insulin in response to physiological glucose levels, as well as secretion of C-peptide and expression of pancreas-specific genes PDX1, Nkx2.2, HLXB-9, and Glut-2 [131, 132]. Later on, Wu and colleagues [133] compared the differentiative ability of WJ-MSCs and BMMSCs in obtaining an IPCs phenotype. Both cellular types were able to form islet-like clusters on the first day in a preconditioned culture medium. In addition, the researchers found a higher expression of PDX1 in differentiated WJ-MSCs compared with the differentiated BMMSCs. Secretion of insulin and mRNA expression of insulin and C-peptide were higher in the differentiated WJ-MSCs [133].

In a study by He et al., WJ-MSCs, infected with PDX1 gene carrying recombinant adenovirus and then treated with inductive factors, could differentiate into IPCs in vitro. They showed that the differentiated cells expressed *β*-cell related genes like PDX1, Ngn3, Glut2, and Nkx6.1 and were able to respond to high concentrations of glucose [134].

In another study, Tsai et al. demonstrated that WJ-MSCs differentiate into IPCs through a three-stage inductive protocol. They also showed that *β*-cell related genes were expressed in both differentiated cells and *β*-like cells transplanted into the liver of STZ-induced diabetic rats through portal vein. As a result, blood glucose levels were significantly reduced 4 weeks after transplantation [135].

Wang et al. could also differentiate WJ-MSCs into IPCs with an inductive medium. They evaluated differentiated cells in vitro in response to the glucose challenge test. After retroorbital injection of IPCs into NOD mice they found that IPCs containing human C-peptide and human nuclei were located in the liver. They concluded that differentiated IPCs from human WJ-MSCs could alleviate hyperglycemia in diabetic mice [48]. These promising data suggest that WJ-MSCs possess the ability, both in vitro and in vivo, to differentiate into insulin-secreting cells.

Due to more similarities of hUCB-MSCs, hPDMSCs, and WJ-MSCs to embryonic stem cells (ESCs) [127, 136, 137], these groups of MSCs should be considered as potential cell therapy options rather than BMMSCs.

With respect to the outstanding differentiation and immunomodulatory capacities of WJ-MSCs, conducting a banking system for both autologous and allogenic transplantation of these cells should be taken into consideration.

### 6.6. Pancreatic Stem Cells

It has been suggested by several studies that pancreatic stem or progenitor cells existing within pancreatic duct cells are able to differentiate and migrate to form new islets during both organogenesis and regeneration. However, it is worth asking whether these cells are MSCs or not. Seeberger et al. [138] demonstrated that stem cells isolated from pancreas can differentiate into osteogenic, chondrogenic, and adipogenic lineages as well as expressing PDX1, Pax4, and Ngn3 transcription factors. Their preliminary data also suggest that these cells have the potential to derive beta cells. Another group [139] successfully isolated pancreatic stem cells from adult human pancreatic duct; these cells not only express nestin and PDX1 but also exhibit the identical markers of MSCs. In an earlier study by Gershengorn et al. [140], it was shown that fibroblast-like cells residing in pancreas are multipotent cells capable of reversible endoderm mesoderm transition (EMT) just like MSCs. However, further research revealed that EMT does not underlie the appearance of fibroblast-like cells in mouse islet cultures but that fibroblast-like cells appear to represent MSC-like cells akin to MSCs isolated from bone marrow [141].

Although there are some restrictions for harvesting pancreatic MSCs, more studies should be done to reveal the underlying mechanisms of their differentiation into beta cells and consequently suggest better differentiation protocols.

## 7. Immunomodulatory Properties of MSCs

Mesenchymal stem cells have been shown to have presumptive plasticity potential to differentiate into multiple lineages and their ability to escape immune recognition and immunomodulatory potentials have received great interest in regenerative and transplantation medicine [46, 142–144]. To date, evidences show that MSCs have immunomodulatory effects on T lymphocytes (T cells), B lymphocytes (B cells), dendritic cells (DCs), and natural killer cells (NK cells). Although the exact underlying mechanism of such effect has not been fully elucidated, these intriguing properties make MSCs a new alternative therapy for autoimmune disorders such as T1DM.

### 7.1. MSCs Interaction with T Cells

Multiple in vivo and in vitro studies have illustrated that MSCs can impair proliferative activities of T cells as well as their function [66, 67, 75, 145–158]. MSCs express surface adhesion molecules, such as VCAM, ICAM-1, ALCAM, and LFA3, and a number of integrins, which are essential for the interaction with T cells [57, 60, 61]. MSCs do not express MHC class II and other costimulatory molecules, such as CD80 (B7-1), CD86 (B7-2), CD 40, and CD 40L that induce T cells anergy [8, 55, 66, 75, 159, 160]. MSCs inhibit the cyclin D2 expression irreversibly and arrest T cells in G0/G1 phase of the cell cycle [151, 161]. Anergic state can be reversed partially with the addition of exogenous IL-2 [162]. MHC class II can be expressed on MSCs surface in presence of INF*γ* in inflammatory conditions [55, 155]. In spite of MHC class II antigen expression and IL-2 addition, MSCs can inhibit allogeneic T cells proliferation in mixed lymphocyte cultures [66, 75, 145, 150, 160].

Several studies revealed that MSCs increase the number of CD4+ and CD25+ regulatory T cells, favored Foxp3 and CTLA4 expression, and suppress function of other T cells subpopulations [67, 81, 152, 163]. Beyth et al. showed that depletion of CD25+ cells from the purified CD4+ T cells did not prevent MSC-mediated inhibition [156].

Many studies have shown that the immunomodulatory effects of MSCs are mediated by soluble factors. These factors include TGF-*β*, HGF [66, 164], PGE2 [67], and IL-10 [94, 142, 156]. Nicola et al. demonstrated that inhibition of TGF-*β* and HGF restores T cells proliferation [66], although contrary evidences showed that supernatants of MSCs were unable to suppress proliferation [60, 142]. However, another study that used semipermeable Transwells system revealed contrary results [165].

It was illustrated that MSCs express indolamine (IDO) protein and exhibit functionally active IDO after stimulation with INF*γ* that catalyzes the conversion from tryptophan to kynurenine and inhibits T cell proliferation [146, 154, 166–170]. In contrast, another study showed that addition of tryptophan or usage of IDO inhibitor did not reverse suppressive effects of IDO [75]; therefore they exhibited that IDO and tryptophan depletion have partial roles in MSCs immunomodulatory effects [166]. In addition, production of PGE2 has been found to be one of the probable soluble factors that MSCs secrete to inhibit T cells proliferation [67]. However, PGE2 inhibition in MSCs and mitogen activated PBMCs coculture or MSCs and antibody stimulated PBMCs coculture was contradictory [75, 151].

Expression of other factors such as IL-10 [156], nitric oxide (NO) [171], HLA-G [163, 172, 173], heme oxygenase-1 [142, 154, 174], and stromal cell-derived factor-1 (SDF-1) [175] by MSCs has shown some effects on their immunosuppressive characteristics.

However, some controversies still remain in this regard. One explanation is that the effect of soluble factors on MSC-induced immunosuppression depends on the type of stimuli received by MSCs (e.g., allogeneic determinants, membrane-bound proteins, mitogens, and cytokines).

Krampera et al. demonstrated that the cell contact is required for MSCs inhibitory effects and inhibitory activity was abrogated when MSCs were added to the T cell cultures in a Transwells system or when MSCs were replaced by MSCs culture supernatant [145]. Augello et al. showed that the B7-H1/PD-1 pathway was involved in cell contact mechanism [176].

### 7.2. MSCs Interaction with Dendritic Cells (DCs)

Dendritic cells (DCs) are the most important antigen-presenting cells [177]. Several studies have demonstrated that MSCs can inhibit differentiation, maturation, and the function of DCs [67, 156, 157, 178]. Some studies showed that MSCs suppress monocyte-derived DCs differentiation with cell contact mechanism [157, 178]. However, other studies confirmed that, at a higher MSC/monocyte ratio, DCs differentiation can also be suppressed by soluble factors [67, 156, 157, 179, 180].

MSCs have been found to suppress expression of HLA-DR, CD1a, CD40, CD80, and CD86 during DCs differentiation and also suppress CD40, MHC class I, and the maturation marker CD83 during the maturation process of DCs [152, 156, 157, 164]. Moreover, MSCs reduced endocytosis capability and IL-2, IL-12, and TNF-*α* production of DCs, as well as increasing secretion of IL-10 and the ability to stimulate T lymphocyte proliferation [67, 156, 157, 178]. MSCs can also reverse the mature DCs into an immature phenotype [157].

Another study showed that MSCs coculture with DCs inhibits antigen presentation and DCs migration to attractive chemokines [179, 181].

### 7.3. MSCs Interaction with B Cells

In addition to T cell suppression, proliferation of stimulated B cells was also suppressed by MSCs [151]. Deng et al. showed that after coronary artery ligation MSCs could effectively inhibit the proliferation of B cells in a dose-dependent manner [182]. But at very low doses stimulating effects were observed [182, 183]. MSCs also decrease B cells activation and immunoglobulin secretion [182, 183]. B cell proliferation was arrested in the G0/G1 phase of the cell cycle by MSCs similar to T cells. One of the MSCs major mechanisms of B cell suppression is soluble factors. MSCs significantly downregulated CXCR4, CXCR5, and CCR7 chemokine receptors of B cells [183].

### 7.4. MSCs and Natural Killer (NK) Cells

Some studies demonstrated that MSCs are not recognized by NK cells [149]. Also, it has been reported that MSCs inhibit the proliferation of NK cells in a dose-dependent manner [151, 152, 166, 184]. Spaggiari et al. showed that MSCs suppress the proliferation of resting NK cells in response to IL-2 or IL-15 [185]. It has been also demonstrated that, in contrast to freshly isolated NK cells, activated NK cells by IL-2 that produced INF*γ* could efficiently lyse autologous and allogeneic MSCs [61, 149]. After upregulation of MHC I expression in MSCs by INF*γ* treatment, MSCs failed to induce NK cells to produce INF*γ* so that the cytolytic activity was reduced [164, 185].

### 7.5. In Vivo Studies of Immunomodulation

Intravenous (IV) injection of MSCs was shown to prolong the survival of allogeneic skin graft in baboons [147]. Allogeneic MSCs could also engraft in bone without rejection in immunocompetent mice, but lymphocytic infiltration was seen at the periphery of the newly formed bone and inside the cartilaginous matrix [148]. IV administration of MSCs can ameliorate new onset encephalomyelitis (EAE) in the model of multiple sclerosis (MS) [159, 186]. Augello et al. reported that the occurrence of severe, irreversible damage to bone and cartilage in model of rheumatoid arthritis was prevented with a single injection of allogeneic MSCs [187]. In contrast, another group explained that MSCs did not have beneficial effect on this model [188]. In dystrophic experimental model, allogeneic and xenogeneic MSC infusions lead to muscle regeneration without host immune response [189]. Saito et al. [190] detected infused xenogeneic MSCs in bone marrow of immunocompetent rats undergoing coronary artery ligation. In other experimental studies Brdu labeled MSCs were detected at lesion territory in traumatic brain injury rat models [191, 192]. MSC infusion increased the success rate of the clinical outcome after hematopoietic stem cell transplantation (HSCT) [193]. Phase I clinical trials revealed the feasibility and safety of MSCs use in HSCT [194]. Le Blanc et al. showed that injection of MSCs improved the clinical outcome in the patient with treatment-resistant grade IV acute graft-versus-host disease (GVHD) of the gut and liver [195]. Moreover, many studies have demonstrated that MSCs were valuable choice for allogeneic HSCT in patients suffering from acute GVHD [76, 196–198]. Contrarily, some studies reported that allogeneic MSCs trigger memory T cells, CD8+T cells, natural killer T (NKT), and NK infiltrating cells response and are thought to be immunogenic [199, 200].

In patients with Hurlers' syndrome, infused MSCs did not provoke alloreactive T cells response or GVHD [201].

Moreover, it has been revealed in phases I and II clinical trials that MSCs are a safe, feasible, and valuable option in treatment of Crohn's disease and its complications [202–205].

Several experimental studies demonstrated that allogeneic or syngeneic BMMSCs could prevent or revert autoimmune diabetes in diabetic animals [99–101, 206–209].

Bassi et al. reported that administrated AD-MSCs reversed hyperglycemia in NOD mice. The underlying mechanisms were induction of regulatory T cells and reduction of CD4+ Th1 response as well as decrease in INF*γ* levels [210].

In a type 1 diabetes model, combination of allogeneic MSCs and sex-mismatched bone marrow cells resulted in normalization of blood glucose and serum insulin levels by T cells suppression [211].

Madec et al. revealed that MSCs could induce IL-10-producing regulatory T cells and suppress beta-cell-specific T cell responses in vitro and in NOD mouse [212].

Various and contrary outcomes might be related to different inflammatory microenvironments, different MSCs isolation and expansion methods, and use of non-well-characterized cells. Furthermore, in different microenvironments, MSCs utilize different mechanisms to exert immunosuppressive function.

Thus, it should be taken into consideration that animal models are not exactly equal to their human counterparts and, in different species, MSCs have different functions [213].

However, according to the contrasting results, more studies should be carried out before MSCs are applied as a therapeutic modality for T1DM.

## 8. MSCs Transplantation Routes

In experimental studies using MSCs for the purpose of glycemic control in diabetic animal models, various routes have been utilized for cell transplantation.

Banerjee et al. [214] and Sumi et al. [215] showed that transdifferentiation of MSCs into IPCs is feasible by intravenous injections. Ezquer et al. [100] observed that BMMSCs given intravenously contribute to the regeneration of the pancreas and kidney in an animal model of T1DM.

In two individual studies, despite different transplantation routes, similar results were obtained. In both studies, rat BMMSCs were successfully differentiated to islet-like cells and could alleviate hyperglycemia in diabetic rats after being transplanted subcutaneously [107] or given through portal vein [216]. Direct injections into pancreas [109], liver [131, 217], and orbital plexus [122], intracardiac infusion [99], and injection under the renal capsule [218] are the other ways introduced as MSCs delivery routes in diabetic models. In some clinical trials, also, MSCs were transplanted via intra-arterial route [219].

Liver is currently the site of choice for clinical islet transplantation, but due to its anatomic, immunologic, and physiologic properties it does not provide the optimal microenvironment for islet cells homing [220–223].

Another site that can be also considered for transplantation is the striated muscle because of its accessibility and angiogenesis potency [136, 224, 225]. Svensson and colleagues showed that muscles provide three times more blood vessels for engrafted islet than renal subcapsular region [226].

However, there is no evidence to confirm which modality is more effective in glycemic control or obtains fewer complications.

To define the best route of transplantation, specific studies should be designed to track MSCs homing.

## 9. Combination Therapy: Is It More Effective Than MSCs Transplantation Alone?

Transplantation of allogeneic or syngeneic BMMSCs, alone or in combination with hematopoietic stem cells (HSCs), was performed on both STZ-induced diabetic mice and NOD models, improving glycaemia and renal lesions as the results [99, 216]. More recently, in a model of murine STZ-induced diabetes, blood glucose and serum insulin levels were normalized following concomitant transplantation of BM cells with syngeneic or semiallogeneic MSCs, via a single injection. In this study, regeneration of recipient-derived pancreatic insulin-secreting cells was attributed to the immunosuppressive effects of MSCs on the beta-cell-specific T lymphocyte response [211]. Urbán et al. also indicated that neither BMCs nor MSCs transplantation was effective alone. They also illustrated that no donor-derived beta cells were found in the recovered animals so that successful treatment of diabetic animals was not due to the reconstitution of the damaged islet cells from the transplant but due to graft initiated endogenous repair process as well as disappearance of beta-cell-specific T lymphocytes from diabetic pancreas [211].

It has been also shown that combined transplantation of BMMSCs and the islets improved the graft function, probably because of revascularization enhancement by MSCs [227].

Controversial results in these studies might be due to their different designs, cell expansion methods, and the characterized cells transplanted in the animal models of diabetes mellitus.

## 10. Single or Multiple MSCs Transplantation: Which Is More Effective?

Banerjee et al. [214] successfully treated STZ-induced diabetes with multiple infusions of BM cells but did not suggest a mechanism for the recovery. Furthermore, Lee et al. [99] reported that repeated transplantation of human MSCs could induce pancreatic islets and renal glomeruli repair in NOD/SCID mice suffering from STZ-induced diabetes. In a more recent study by Ho et al. [228], it was demonstrated that, compared to single intravenous transplantation, which only transiently decreased hyperglycemia, multiple MSC transplantations effectively restored blood glucose homeostasis. In their study, multiple human MSC transplantations (4.2 × 10^7^ cells/kg each time) were performed intravenously at 2-week intervals into STZ-induced diabetic mice for 6 months. They described that multiple transplantations were essential to restore and maintain glucose homeostasis through decreasing systemic oxidative stress in the early stage and insulin production in the late stage.

However, according to our unpublished clinical trial results, repeated MSCs administration in patients with no response to the first intravenous transplantation revealed no advantages.

Thus, before the second course of transplantation, diabetic patients general condition as well as their immunological state must be evaluated and the optimum dose of MSCs should be defined.

## 11. Are Diabetic Patients' MSCs Suitable Candidates for Treatment of Diabetes?

In a study by Dong et al. [229], allogenic diabetic MSCs transplanted intravenously could initiate endogenous pancreatic regeneration by neogenesis of recipient islets. Thus, it was concluded that diabetic MSCs retain their stemness and potential to induce pancreatic regeneration on transplantation.

Moreover, Sun et al. [230] showed that BMMSCs from (both type 1 and type 2) diabetic patients also can differentiate into IPCs under appropriate conditions (18 days, three-stage protocol) in vitro. Their results provide the direct evidence for the feasibility of using patients' own BMMSCs as a source of IPCs for beta cell replacement therapy.

Some studies stated that prolonged exposure to hyperglycemia facilitates and enhances MSCs differentiation potency to IPCs [110, 218, 231, 232]; however, BMMSCs yielding in addition to their potency decreases throughout the life in normal population. Moreover, several studies have shown that hyperglycemic state and underlying defective microenvironment in diabetic patients impair MSCs function [46, 106, 233–236]. Therefore, considering another source of MSCs for cell based therapies in diabetic patients seems to be of great advantage.

In addition, duration of diabetes and MSCs potency are other important issues that must be taken into account before stem cell transplantation in a patient.

## 12. Role of Genetic Manipulation in MSCs Administration for Treatment of Diabetes Mellitus

Xu et al. [217] showed that murine BMMSCs harboring human insulin gene could relieve diabetes for up to 6 weeks by intrahepatic administration in animal models.

Boumaza et al. [206] demonstrated that autologous BMMSCs can be administered to promote PDX1 and insulin protein expression locally in the pancreas and provide sustained systemic levels of insulin to treat or cure diabetes in the STZ-induced diabetic rats. Thus, blood-derived autologous MSCs can be carefully generated ex vivo and infused prior to the onset of full-blown diabetes to prevent beta cell damage and to modulate autoreactive T cell activation, while avoiding potential complications and cost for islet transplantation and long-term immunosuppression.

Lin et al. [237] revealed that rat BMMSCs transplantation followed by IV injection of recombinant lentiviruses encoding 2 different small hairpin RNAs (shRNAs) for specific interference with neurogenin 3 (Ngn3) could lower blood glucose by increasing beta cell mass compared with sham operated controls.

In the same year, Zhu et al. [238] demonstrated that experimental diabetes in beagle dogs could be relieved effectively for up to 16 weeks by intrahepatic autotransplantation of BMMSCs expressing human insulin. In a study by Moriscot et al. [103] it was indicated that by infection with adenoviruses coding for several transcription factors of the beta cell developmental pathway and coculture with islet tissue or islet-conditioned medium hBMMSCs are able to differentiate into insulin-expressing cells. In two other studies [218, 231] it was presented that pancreatic duodenal homeobox 1 (PDX1) gene-modified hBMMSCs can be differentiated into functional IPCs. Karnieli et al. [218] transfected MSCs with PDX1 and transplanted them under the renal capsule of STZ-diabetic SCID mice. After 5 weeks, glucose levels reduced from above 300 to 200 mg/dL. However, 6–8 weeks after transplantation an abnormal response to the glucose tolerance test was noted.

In a more recent study in 2012, Milanesi et al. [239] examined the ability of hBMMSCs genetically modified to transiently express VEGF or PDX1 to reverse diabetes. As a result, hBMMSCs expressing VEGF and PDX1 reversed hyperglycemia in more than half of the diabetic mice and induced overall improved survival and weight maintenance in all mice. However, recovery was sustained only in the mice treated with hBMMSCs-VEGF. Furthermore, de novo beta cell differentiation from human cells was observed in mice in both cases, treated with either hBMMSCs-VEGF or hBMMSCs-PDX1.

Taken collectively, MSCs can be differentiated into IPCs by genetic manipulation, but whether this strategy is a safe modality in clinical applications needs further investigations.

Hence, there are still serious concerns about the safety and the efficacy of manipulated cells as well as unresolved ethical issues regarding their use in the prospective gene therapy.

## 13. MSCs and Biomaterial Composites in Diabetes

In islet transplantation approach, one potential strategy to overcome immune rejection and lifelong use of immunosuppressive drugs is islet encapsulation with semipermeable biomaterials [240–242]. Immunoprotective biomaterials can be used in islet encapsulation to create a permselective membrane around a group of islet cells. This type of device is called a bioartificial pancreas [242]. Despite the improvement of islet cell survival and efficacy rates, one of the obstacles is oxygenation limitation which results in loss of islets [241, 243–246].

There are some studies that used various biomaterials for encapsulation of or a scaffold for islet beta cells, ESCs-derived IPCs, and even xenogeneic islet cells [242, 243, 246–252].

Some other studies used the composition of biomaterials and MSCs in treatment of diabetes complications.

Chandra et al. encapsulated AD-MSCs-derived insulin-producing aggregates (ICAs) with alginate calcium and then transplanted them into peritoneal cavity. After 2 weeks, diabetic mice retained normoglycemic state. After 4 weeks, the transplanted capsules were removed, and ICAs showed cellular integrity, viability, and functionality [120].

In another study, Ngoc et al. firstly differentiated mouse BMMSCs and human UCMSCs into IPCs under three-step inductive protocol. In the next step they encapsulated IPCs in an alginate membrane. Finally they transplanted these capsules into peritoneal cavity. Simultaneously, they transplanted unencapsulated IPCs into diabetic mice through intraportal route. They showed that allogeneic and xenogeneic encapsulated IPCs had more survival than IPCs alone. Also encapsulation prevents immune response to xenogeneic and especially to allogeneic IPCs. Moreover hyperglycemic state improvement was observed in both xenogeneic and allogeneic encapsulated IPCs recipients [253].

Vériter et al. covered porcine islet cell scaffold composite with MSCs and demonstrated that MSCs can improve oxygenation of encapsulated islet cells with an increase of vascularization in the periphery of the bioartificial pancreas [254].

However, cotransplantation of MSCs and biomaterials, with the aim of preparing a more appropriate tridimensional microenvironment for beta cells transplantation and preventing immune rejection, should be evaluated more before being applied in cell-based therapies.

## 14. Application of MSCs in Human Studies

Although MSCs have tremendous therapeutic potentials, limitations such as lack of a standardized protocol to expand them, poor engraftment, limited differentiation under in vivo conditions, malignant transformation, and unwanted release of cytokines need to be overcome to enhance their clinical utility [5].

Risk of tumor induction is the issue that should be always taken into great consideration. Spontaneous malignant transformation of MSCs [209, 255–257] and promotion of tumor development and growth [148] are possible mechanisms accounting for their tumor formation potential.

In contrast to in vitro studies, spontaneous transformation has not been noted in clinical trials using human MSCs to date [256, 257]. The significant inhibitory effects of MSCs on the proliferation and function of major immune cells populations may also play an important role in favoring cancer development and/or progression, as well as in helping preexisting non-MSC-derived tumors escape immune surveillance [148, 258, 259].

However, there are several clinical trials under way that might reveal the safety of MSCs administration in type one diabetic patients in the near future.

Trivedi and colleagues administrated ADSCs-derived IPCs, prepared in xenogeneic-free condition, in combination with HSCs into 11 insulin dependent patients without any immunosuppressive agents. Their study revealed promising results such as reduction in exogenous insulin requirement, increase in C-peptide levels, and having no episodes of diabetic ketoacidosis and host immune response. None of patients showed any unwanted effects [260].

Vanikar et al. cotransplanted AD-MSCs-derived IPCs and cultured bone marrow intraportally to 11 conditioned patients who irradiated and received anti-T and anti-B cells antibodies. They followed up patients for 23 months and exogenous insulin requirement and HbA1c level of patients decreased over this period, but insulin independency occurs in any patient. C-peptide give raised over follow up and any unwanted effects and ketoacidosis did not observe in patients [261]. However, complete insulin independency did not occur in their study, but safety and effectiveness of ADSCs-based cell therapy in diabetes have been shown. Thus, longer follow-ups must be done to demonstrate long-term safety and efficacy of the administered cells.

In another clinical study done by Hu et al., long-term efficacy of IV administration of WJ-MSCs in 15 new onset type 1 diabetic patients was assessed. They followed up the patients for their insulin requirements and HbA1c and C-peptide levels for 24 months after transplantation. Three patients became insulin independent in the end of the follow-up period. In other patients, insulin requirements and HbA1c levels decreased significantly in comparison to the control group. Also, C-peptide levels increased significantly in patients undergoing WJ-MSCs transplantation. They did not report any acute or chronic side effects or ketoacidosis in transplantation group while in control group ketoacidosis appeared in 3 patients [262].

Chen and coworkers infused autologous bone marrow mononuclear stem cells (including MSCs and HSCs) into pancreas through splenic artery. They reported beneficial effects of stem cell transplantation on insulin requirements and HbA1c and C-peptide levels of the patients. The authors have not published the whole data yet [263].

In our unpublished clinical study, we transplanted autologous BMMSCs intravenously to new onset type 1 diabetic patients without any immunosuppressive therapy. After 3 months, the patients who still required exogenous insulin to maintain blood glucose levels in normal ranges received another infusion. Six months after the first infusion, two patients became insulin independent and, in some patients, beneficial effects on their insulin requirements and C-peptide and HbA1c levels were seen. The rest of the patients did not benefit from the transplantations. However, no acute or chronic complications related to MSCs transplantation have been observed in the patients so far.

There are also some ongoing clinical trials (registered at https://www.clinicaltrial.gov/) in which MSCs from autologous or allogeneic sources are being transplanted to patients with T1DM (Table 1).

For instance, in Osiris clinical study, investigators are performing allogeneic MSCs transplantation on patients with T1DM to assess its safety and efficacy.

## 15. Conclusion

Although islet transplantation has opened new horizons in the treatment of T1DM, scarcity of supply, long-term immunosuppressive therapies, and rejection of grafted cells have restricted its application in the clinical setting. MSCs, as discussed previously, have great immunomodulatory and multilineage differentiation capacities. According to their accessible sources, it can be assumed that there would be unlimited sources for IPCs. However, there are still serious concerns about clinical application of MSCs.

One of the important issues regarding MSCs transplantation is the adequate number of differentiated or undifferentiated cells necessary to keep plasma glucose levels within normal ranges. However, sustained euglycemia might be disproportionate to the number of injected cells due to MSCs transdifferentiation into IPCs in vivo [264].

Another important issue is the evaluation of immunomodulatory effects of differentiated cells. It should be discussed more clearly at which stage of MSCs differentiation into IPCs they retain their immune suppressive effects. It has been suggested that undifferentiated MSCs can be used in T1DM treatment based on their immunosuppressive properties [142].

Minimum or no application of immunosuppressive drugs is favorable for MSCs or IPCs derived from MSCs because of their attenuating effects on the cells or their fatal complications in the recipients [136].

Nevertheless, more investigations are necessary to define the minimum dosage of immunosuppressants which does not alter the differentiation potency of combined MSCs to prevent even minor immune system response in the recipients.

One other concern about undifferentiated MSCs is their differentiation potency to unwanted tissues. Long-term cultivations and more cell passages to improve differentiation efficacy of MSCs can induce mutations and cell transformations [257].

According to some studies done on murines, MSCs, especially in allogenic transplantation, might lead to tumor formation [148, 265, 266]. However, contrary studies revealed that MSCs inhibit tumor growth both in vitro and in vivo [267, 268].

Another issue, expected to be clarified soon, is the well characterization of markers expressed by MSCs to prevent immunological response in recipients. In addition, using autologous serum or serum-free and xenogeneic-free culture conditions should also be taken into account particularly in the clinical settings because fetal bovine serum (FBS), instead, can induce immune and allergic response especially in repeated administrations [269].

For broad application of MSCs in treatment of T1DM in the future, more clinical studies with various sources of MSCs, larger population of patients undergoing transplantation, and longer monitoring duration are required to determine safety and efficacy of this novel therapeutic approach. Additionally, utility of biomaterials, growth factors, and improvement of oxygen carriers should also be evaluated in further studies.

## Figures and Tables

**Table 1:** 

Study	Year	Identifier	Site/principal investigator	Intervention	Status/enrollment
“Treatment of Patients with Newly Onset of Type 1 Diabetes with Mesenchymal Stem Cells Phase I/II”	2010	NCT01068951	Uppsala University Hospital	Autologous transplantation of the patients' own mesenchymal stem cells (approximately 2 × 10^6^ cells/kg body weight) intravenously	Completed 2013; 20 patients

“PROCHYMAL (Human Adult Stem Cells) for the Treatment of Recently Diagnosed Type 1 Diabetes Mellitus (T1DM) Phase I”	2008	NCT00690066	Mesoblast International Sàrl	Intravenous infusion of ex vivo cultured adult human mesenchymal stem cells (PROCHYMAL)	Completed 2011; 63 patients

“Umbilical Mesenchymal Stem Cells and Mononuclear Cells Infusion in Type 1 Diabetes Mellitus Phase I/II”	2011	NCT01374854	Fuzhou General Hospital	Infusion of 1 × 10^6^/kg UCMSCs through pancreatic artery along with mononuclear cells by interventional therapy and another administration of same dose of UCMSCs one week after intervention	Active, not recruiting; 44 patients

“Autologous Transplantation of Mesenchymal Stem Cells for Treatment of Patients with Onset of Type 1 Diabetes Phase II/III”	2010	NCT01157403	Lu Debin, Third Military Medical University	Autologous transplantation of bone marrow mesenchymal stem cells (approximately 2.5 × 10^6^ cells/kg body weight) intravenously	Recruiting; 80 patients

“Safety and Efficacy of Mesenchymal Stem Cells in Newly Diagnosed Type 1 Diabetic Patients Phase I/II”	2010	NCT01322789	University of Sao Paulo	Intravenous mesenchymal stem cell infusion; four consecutive intravenous infusions 1 week apart followed by 4 consecutive infusions 1 month apart	Recruiting; 10 patients

“Cotransplantation of Islet and Mesenchymal Stem Cell in Type 1 Diabetic Patients Phase I/II”	2008	NCT00646724	Fuzhou General Hospital	Cotransplantation of islet and mesenchymal stem cell; islet of allograft and MSCs of autograft	Recruiting; 30 patients

“Human Menstrual Blood-Derived Mesenchymal Stem Cells Transplantation in Treating Type 1 Diabetic Patients Phase I/II”	2011	NCT01496339	S-Evans Biosciences Co., Ltd.	1 × 10^6^/kg MenSCs are infused through pancreatic artery or intravenous infusion once a week by the 4 consecutive therapies	Recruiting; 50 patients

“Stem Cell Therapy for Type 1 Diabetes Mellitus Phase I/II”	2010	NCT01143168	Cellonis Biotechnology Co.	First transplantation: on day 0, ABM-MNCs + UCMSCs through pancreas artery; second transplantation: on day 7 ± 1, ABM-MNCs + UCMSCs intravenously; third transplantation: on day 14 ± 2, ABM-MNCs + UCMSCs intravenously	Active, not recruiting; 24 patients

“Mesenchymal Stem Cells to Intervene in the Development of Type 1 Diabetes: A Blinded Randomized Study Phase II”	2014	NCT02057211	Uppsala University Hospital	Autologous mesenchymal stem cell transplantation	Recruiting; 50 patients

ABM-MNCs: autologous bone marrow mononuclear cells, UCMSCs: umbilical cord mesenchymal stem cells, and MenSCs: menstrual blood-derived mesenchymal stem cells.
